# The State of Play with iPSCs and Spinal Cord Injury Models

**DOI:** 10.3390/jcm4010193

**Published:** 2015-01-14

**Authors:** Stuart I. Hodgetts, Michael Edel, Alan R. Harvey

**Affiliations:** 1 School of Anatomy, Physiology and Human Biology, The University of Western Australia, Crawley, Western Australia 6009, Australia; E-Mail: alan.harvey@uwa.edu.au; 2 Control of Pluripotency Laboratory, Department of Physiological Sciences I, Faculty of Medicine, University of Barcelona, Hospital Clinic, Casanova 143, Barcelona 08036, Spain; E-Mail: edel.michael@gmail.com; 3 Faculty of Medicine, The University of Sydney Medical School, Division of Pediatrics and Child Health, Westmead Children’s Hospital, Sydney 2010, Australia; 4 School of Anatomy, Physiology and Human Biology, and the Harry Perkins Institute for Medical Research (CCTRM), The University of Western Australia, Western Australia 6009, Australia

**Keywords:** spinal cord injury, induced pluripotent stem cells, transplantation

## Abstract

The application of induced pluripotent stem cell (iPSC) technologies in cell based strategies, for the repair of the central nervous system (with particular focus on the spinal cord), is moving towards the potential use of clinical grade donor cells. The ability of iPSCs to generate donor neuronal, glial and astrocytic phenotypes for transplantation is highlighted here, and we review recent research using iPSCs in attempts to treat spinal cord injury in various animal models. Also discussed are issues relating to the production of clinical grade iPSCs, recent advances in transdifferentiation protocols for iPSC-derived donor cell populations, concerns about tumourogenicity, and whether iPSC technologies offer any advantages over previous donor cell candidates or tissues already in use as therapeutic tools in experimental spinal cord injury studies.

## 1. Introduction

Spinal cord injury (SCI) is characterised by damage to sensory and motor function, the extent of any functional loss dependent on the location, extent (severity) and type of injury (contusion *vs.* transection, incomplete *vs.* complete). Sensorimotor loss that results from a primary mechanical injury is a result of many interacting pathological factors, including: axonal damage, loss of neurons, activation of astrocytes and microglia, degeneration of oligodendrocytes, and demyelination [1]. The extent of this initial damage is significantly increased by ensuing secondary cascades of ischaemia, anoxia, generation of damaging free-radicals, lipid peroxidation, excitotoxicity, and immune-mediated and inflammatory events (e.g., cytokines), which can stimulate further cell death and tissue loss. A region of spreading degeneration rostral and caudal to the injury site, together with inhibitor molecule production, eventually leads to cavitation as well as a glial scar rich in, among other things, various types of chondroitin sulphate proteoglycans (CSPG) that are extremely inhibitory to axonal regrowth. Strategies to induce repair and promote functional (locomotor) recovery generally aim to reduce the extent of secondary damage and demyelination, promote the re-myelination of damaged (but still viable) axons, induce axonal repair and/or regeneration, and perhaps stimulate an endogenous stem cell response. For decades, extensive research has been conducted into clinically relevant cell transplantation strategies to either promote regeneration or to replace damaged/missing cell populations using: fibroblasts, peripheral nerve grafts and Schwann cell bridges, olfactory ensheathing glia (OEG), embryonic stem cells (ESCs), oligodendroglial progenitor cells (OPCs), adult neural precursor cells (NPCs) and neural stem cells (NSCs), autologous macrophages and mesenchymal precursor cells (MPCs) isolated from bone marrow stroma (BMSCs) (for reviews see [2,3,4,5]). More recently, the possibility of developing strategies that use induced Pluripotent Stem Cell (iPSC) technology to generate donor cell populations has gathered momentum.

## 2. iPSCs as Neuronal and Glial Candidate Donor Populations

To date, iPSCs have been directed to generate neural crest cells [6,7], peripheral sensory neurons [8], neural stem cells and their neuronal progenitors including specific neuronal subtypes such as dopaminergic neurons [9,10,11,12,13,14,15,16,17] glutamatergic neurons [18,19,20,21], GABAergic neurons [18,19,22], motor neurons [23,24,25,26,27,28,29] (see also Faravelli *et al.* 2014 for review of methodologies of induction into motor neurons [30]), retinal neurons [31,32,33,34], as well as astrocytes [35,36,37,38] and oligodendrocyte lineages [37,39,40,41,42,43]. iPSCs and their derivatives have been tested in various *in vivo* animal models of neurological/neurodegenerative disorders including Parkinson’s Disease [9,10,11,12,14,44], demyelination [37,39,40,41,42,43], retinal regeneration [32,33], stroke [45,46,47,48] and peripheral nerve regeneration [7] as well as others (see [49,50]). These studies provide proof-of-principle that iPSCs can be successfully differentiated *in vitro* to yield a desired progeny that, if necessary, can be effectively subjected to *ex vivo* gene therapy [51,52] and then transplanted with similar outcomes to other pluripotent ESC therapies [53,54,55,56].

## 3. iPSCs in Spinal Cord Injury

Despite a rapid increase in iPSC-based studies in recent years, currently there is only a small number of published preclinical studies describing the *in vivo* use of iPSCs in mouse [57,58,59], rat [36,50,57,58,59,60,61,62] or simian [37,60,61,62] models of SCI, or sub-dural parenchymal injections into non-injured rats [63].

Of these studies, rodent moderate contusion injuries were almost all made at the thoracic level (T9–T10) using the Infinite Horizon Impactor device (delivering 60–70 kDyne forces for mice and 200 kDyne force for rat). An exception was a study that used C4 contusions using the Ohio State Injury Device [61], and Lu *et al.* [60] recently used C5 lateral hemisections in rats. Simian contusions have to date been more severe (17 g 50 mm drop at C5 using the NYU impactor [37] or a 50 g 10 mm drop at T9 [62]). All published studies using contusive SCI (apart from [62]) have reported neuronal, glial and astrocytic marker expression within or near the lesion after transplantation, with two groups reporting differentiation of donor cells into at least one or all these various cell types [37,50,58,59,60,61,62,63]. These studies used iPSC donor cells that were pre-differentiated into either neurospheres (NS) [58,59], neural precursor cells (NPCs) [61,63], neural stem cells (NSCs) [60,61,62] astrocytes [36] or undifferentiated iPSCs [50]. Sareen *et al.* [63] found that NPCs derived from iPSCs showed variability in differentiation phenotype and survival characteristics following transplantation, but migrated and integrated within the uninjured cord. Superparamagnetic iron oxide labelled iPSC-derived NSCs were tracked non-invasively using magnetic resonance imaging (MRI) from the cell injection sites in monkeys that extended progressively to the lesion regions [62]. Transplanted iPSC-derived NPCs after early chronic cervical SCI were shown to form neurons, astrocytes and oligodendrocytes at 8 weeks post transplantation, however importantly failed to promote functional recovery in forelimb behavioural tasks.

Whilst murine SCI studies using iPSC-derived donor cells showed functional improvements, others have reported no significant differences in morphological or functional outcomes in another acute moderate contusion SCI model in rats [36,50,60]. Lu *et al*. [60] reported that 3 months after transplantation, surviving human iPSC-derived NSCs from an 86 year old donor male exhibited extraordinarily long distance axonal growth with the host rat spinal cord, with human axons growing rostral and caudal to the lesion site and forming synaptic structures with host neurons and dendrites. Such extensive growth of immature human cells within the rodent central nervous system (CNS) is similar to that obtained many years ago using grafts of human fetal tissue and neuroblasts (e.g., [64]). In the iPSC study, host axons grew into the donor grafts and also formed synaptic structures, again similar to previous work that used donor fetal material of some kind (e.g., [65]). Taken together the new iPSC work confirms that even in the injured adult CNS it is possible, in some cases, to overcome the inhibitory environment of the lesion and elicit substantial regenerative growth and circuit construction. The grafting technique used by Lu *et al.* 2014 [60] involved a cocktail of growth factors (including brain-derived neurotrophic factor, neurotrophin-3, platelet-derived growth factor-AA, insulin-like growth factor-1, epidermal growth factor, basic fibroblast growth factor, acidic fibroblast growth factor, glial cell line-derived neurotrophic factor, hepatocyte growth factor, and calpain inhibitor in a fibrin matrix) that previously was shown by the same group to promote robust engraftment of donor (non-iPSC derived) NSCs, extensive integration with host tissue, long-distance outgrowth of axons from grafts and extensive ingrowth of host axons into the graft after acute thoracic (T3) SCI [66].

Significant functional improvement was reported in the initial NSC study [66]; however more recently, Lu *et al.* (2014) in a C5 lateral hemisection study [60], reported no measurable improvement in forelimb function in host rats despite the use of the same growth cocktail, extensive axonal outgrowth and cellular integration. The authors suggest that the injury type itself, the rate of maturation of donor cells (so that insufficient numbers of mature neurons were present to support recovery), inadequate myelination, undesirable ectopic projections and/or insufficient expression of neurotransmitters could account for the discrepancy between the functional recovery observed between the two studies. Whilst the extent of hindlimb *versus* forelimb recovery may vary depending on the type and complexity of restored or adapted neural circuitry [60], it is also important to note that independent researchers that attempted to replicate this study (as part of the NIH “Facilities of Research Excellence-Spinal Cord Injury” project to support independent replication) revealed conflicting data relating to ingrowth of host axons into the grafts and behavioural outcomes [67]. Overall, these are very important and influential studies, but the extent to which reported differences also reflect, for example, variation in surgical procedures, the individual contributions of factors in the growth cocktail [68], or differences in the nature and response of the donor cell type after transplantation, needs to be established, and future work should yield valuable information in this regard.

The approach of using restricted or individual populations of donor cells in the hope of achieving regrowth or repair leading to morphological improvements and functional restoration has some limitations. The ability of a wide variety of adult somatic (e.g., Schwann cells, olfactory ensheathing glia) and precursor/progenitor (e.g., NPCs, NSCs, OPCs, MPCs) cells to undergo directed differentiation and perform functionally and phenotypically as required *in vitro* has not always been reproduced when cells are transplanted into the inhibitory environment of the injured spinal cord *in vivo*. Perhaps these well characterised donor cells that meet necessary research requirements in a wide variety of controlled settings other than the injured spinal cord, simply fail to “perform” in animal models *in vivo* because of the antagonistic, often inflammatory environment they find themselves in after transplantation [69]. Those donor cells that eventually survive the host immune response may be unable to successfully respond to the new and dynamic myriad of both inhibitory and growth promoting stimuli of the host’s injured spinal cord that is known to occur in a temporal and spatial fashion after trauma. Simply, the “correct language” that equipped the donor cells with the ability to perform all of those functions observed under controlled conditions *in vitro*, is no longer able to be understood or followed *in vivo*. Perhaps by using combined populations of adult stem cell-derived oligodendrocyte, astrocyte and neuronal precursor cells in the same relative proportions as those found within the uninjured (normal) spinal cord, we may achieve a phenotypic state that will allow enhanced plasticity and optimal repair/regrowth.

It is crucial to ensure that appropriate cell controls are used in preclinical SCI studies to evaluate the extent of contribution of different cell phenotypes to the morphological and functional outcomes observed after treatment. This applies to any small populations of incompletely reprogrammed donor cells and/or incompletely pre-differentiated donor iPSCs. Whilst the studies mentioned may suggest that improved outcomes were observed in mouse but not necessarily in rat models of SCI, the disparity in overall results from these very limited number of studies suggest that iPSC-based therapy in SCI warrants more extensive and thorough testing. Ideally, research in this area should be conducted using clinically relevant injury regimes in at least mouse and rat models as outlined in the recommendations and guidelines developed by the International Campaign for Cures of Spinal Cord Injury Paralysis (ICCP) [70] (see also [71,72]). Experimental studies in larger species (e.g., cats and primates) with an ascending and descending tract configuration more similar to the human [73], and capable of more complex sensorimotor behaviors, should also be undertaken. In addition, it may be important to include more relevant control donor cell types, such as cells that have been freeze-thawed.

## 4. Conclusions

There is a clear need to develop a gold standard positive control for use of stem cells in animal models of SCI, to determine the validity and reliability for future clinical application. It is most likely that stem cell therapy alone will not work for SCI, but will require new efforts to combine stem cell therapy with other treatments perhaps, such as bio-scaffolds, immune response modifications, and the timing of the use of different treatments, although the consensus at present is “the earlier the better”. The threat of tumorigenicity remains to be fully addressed. In SCI studies that used iPSC-derived donor cells, “unsafe” murine iPSC-derived donor cells, but not “safe” donor cells, produced teratomas [59], although another study did not report such teratoma formation [36]. Of those studies using human iPSC-derived donor cells, one study did not report on teratoma formation [57], whilst others reported no evidence of tumour formation [37,50,58,60,61,62,63]. For clinical applications, donor cells must be grown in animal cell-free and serum-free conditions and derivation of the first hESC line with these properties has been a major advance for clinical applications of stem cell therapy [74]. Despite their highly similar expression of genes related to pluripotency and development, there is evidence that iPSCs may occupy a distinct pluripotent “state” from ESCs [50,75], and therefore iPSCs may not have the same capacity as ESCs to generate the whole spectrum of region-specific neural progenitors and functional neuronal subtypes for SCI therapies (and other CNS disorders). Nevertheless, the approaching capacity to produce clinical grade iPSCs, together with advances in the efficiency of transdifferentiation protocols for iPSCs into the required phenotypes, marks a potential focus toward the use of iPSC-derived donor cell populations for cell based therapies. If hESC-derived OPCs can be used in SCI trials (Geron), this should surely herald the addition of the clinical grade iPSCs to the potential repertoire of donor cell candidates for SCI and other neurotrauma related therapies, as long as they are conducted in accordance with Good Clinical Practise (GCP) and the associated regulatory directives.
